# Longitudinal Deterioration in Nutritional Status Associated With Increased Risk of Sarcopenia in Community‐Dwelling Aged Adults: A Prospective Cohort Study

**DOI:** 10.1002/jcsm.70270

**Published:** 2026-04-01

**Authors:** Ho Jun Lim, Seung Shin Park, Mi Ji Kim, Hak Chul Jang, Sung Hye Kong, Chang Won Won

**Affiliations:** ^1^ Department of Internal Medicine Seoul National University Bundang Hospital Seoul Republic of Korea; ^2^ Department of Internal Medicine Seoul National University Hospital Seoul Republic of Korea; ^3^ Department of Family Medicine, College of Medicine, Kyung Hee University Kyung Hee University Medical Center Seoul Republic of Korea; ^4^ Department of Internal Medicine, College of Medicine Seoul National University Seoul Republic of Korea

**Keywords:** AWGS, KFACS, Mini Nutritional Assessment, nutrition, sarcopenia

## Abstract

**Background:**

The association between changes in nutritional status over time and the risk of sarcopenia in older adults is unclear. This study investigated the impact of baseline nutritional status and its 4‐year changes on the development of sarcopenia and severe sarcopenia.

**Methods:**

We conducted a prospective, multicentre longitudinal cohort study and 1661 community‐dwelling adults aged 70–84 years without baseline sarcopenia were included for analysis. Nutritional status was assessed using the Mini Nutritional Assessment (MNA), categorized as normal, at risk of malnutrition or malnutrition (24–30, 17–23.5 and < 17 points, respectively). Changes in nutritional status were classified as improved, unchanged or deteriorated. Primary outcomes were new‐onset sarcopenia and severe sarcopenia. Sarcopenia was defined by the Asian Working Group for Sarcopenia (AWGS) 2019 criteria. Cox regression models using changes in nutritional status as a time‐varying covariate were used to estimate hazard ratios (HRs), adjusted for age, sex, body mass index, smoking, alcohol use, fall history, education, comorbidities and activities of daily living. In sensitivity analyses, the Fine and Gray subdistribution hazard model was performed to estimate subdistribution HRs (sHRs), which accounts for death as a competing risk.

**Results:**

The mean age of participants was 76.3 ± 3.6 years, and 46.9% were male. At baseline, 77.3% had normal nutritional status, 21.8% were at risk of malnutrition, and 0.9% were malnourished. After mean follow‐up of 6.8 years, deterioration in nutritional status was associated with increased risk of sarcopenia (HR 1.77 [95% confidence interval, 1.25–2.51], *p* = 0.001) and severe sarcopenia (HR 1.97 [1.23–3.16], *p* = 0.005) compared to those with unchanged nutritional status among total participants. Among those with normal nutritional status at baseline, deterioration in nutritional status was also associated with increased risk of sarcopenia (HR 2.07 [95% confidence interval, 1.37–3.12], *p* = 0.001) and severe sarcopenia (HR 1.95 [1.08–3.53], *p* = 0.028). Prevalence of low muscle mass differed significantly by measurement device (Hologic 64.8%, GE Lunar 37.2%, BIA 24.2%, *p* < 0.001); however, the longitudinal association between nutritional status and sarcopenia risk remained consistent in sensitivity analysis.

**Conclusion:**

Baseline nutritional risk and deterioration in nutritional status are robust predictors of future sarcopenia and severe sarcopenia in community‐dwelling older adults. Improving nutritional status over time may significantly reduce the risk of progressing to severe sarcopenia. Clinical strategies to monitor and enhance nutrition are essential for preventing sarcopenia.

## 1 Introduction

Sarcopenia is a progressive and generalized syndrome characterized by age‐related loss of skeletal muscle mass, strength and physical performance [1, 2]. First introduced by Irwin Rosenberg in 1989, sarcopenia has become a growing public health concern due to its association with increased risks of physical disability, poor quality of life and mortality [3]. The prevalence of sarcopenia rises significantly with age, reaching up to 53% in individuals over 80 years old [4]. As global populations continue to age, especially in super‐aged societies such as South Korea, identifying and modifying risk factors of sarcopenia remains a critical challenge for the medical community.

Among the various risk factors of sarcopenia, nutritional status plays a critical role [5, 6]. Malnutrition is recognized as a key contributor to the progression of sarcopenia, exacerbating age‐related physiological changes such as impaired muscle maintenance [7]. Poor nutritional intake aggravates muscle degradation, increasing susceptibility to sarcopenia and its associated complications [8]. Indeed, in previous cross‐sectional studies, poor nutritional status has been associated with a higher risk of muscle loss, strength decline and performance deterioration, thereby increasing the risk of sarcopenia by approximately two to three times [9, 10]. In previous longitudinal studies, poor nutritional status at baseline has also been associated with the development of sarcopenia over time [8]. In addition, from a clinical perspective, understanding whether improvements or deteriorations in nutritional status are associated with the risk of sarcopenia is both important and compelling. However, whether longitudinal changes in nutritional status over time contribute to the risk of sarcopenia has not been thoroughly investigated.

To address this gap, our study aimed to examine the longitudinal association between changes in nutritional status and the risk of sarcopenia among older community‐dwelling adults in a large, multicentre longitudinal cohort.

## 2 Materials and Methods

### 2.1 Study Design and Participants

**FIGURE 1:**
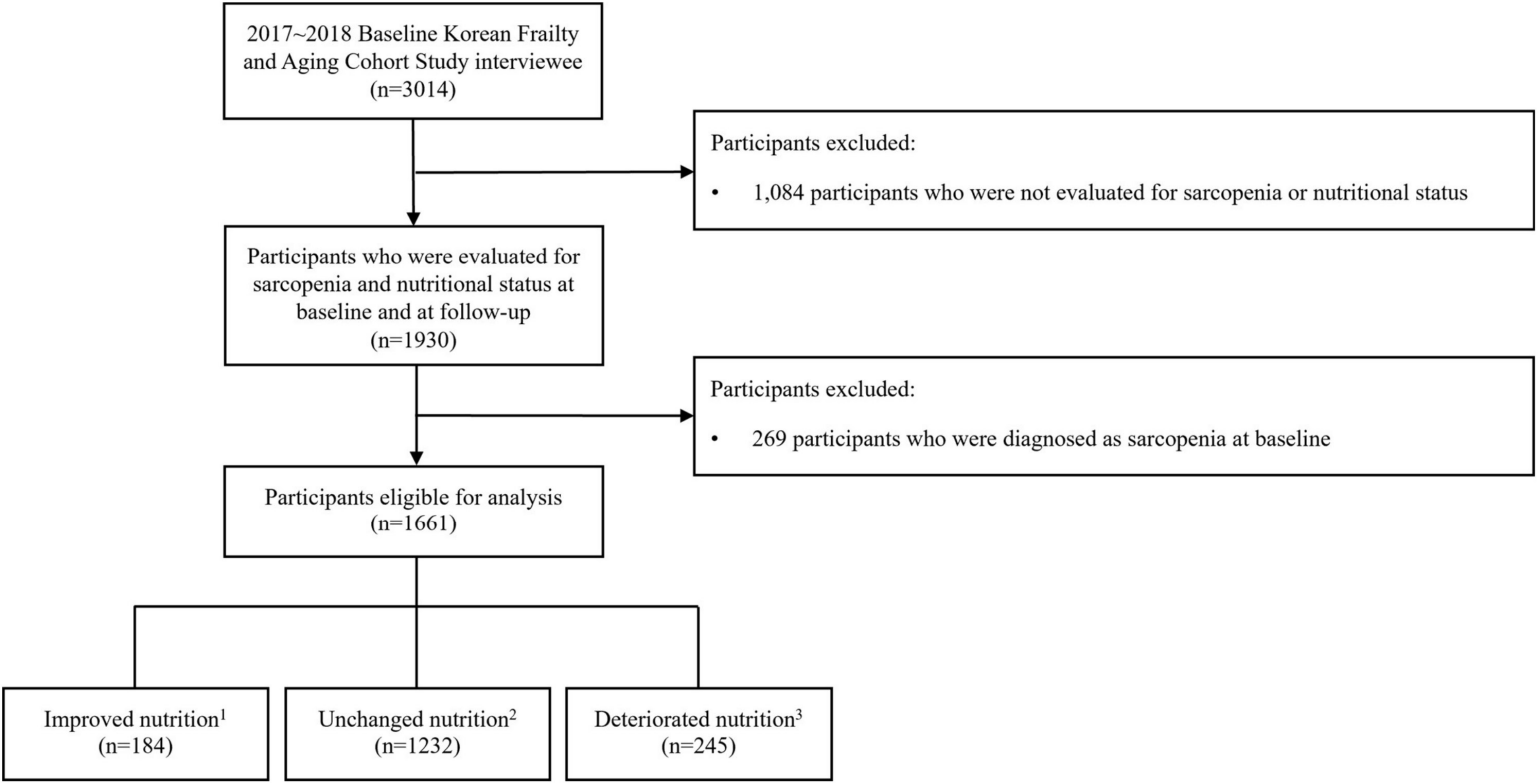
Flow diagram of the inclusion and exclusion of participants. ^1^Improved nutrition: Participants whose nutritional status according to the Mini Nutritional Assessment was improved at follow‐up. ^2^Unchanged nutrition: Participants whose nutritional status according to the Mini Nutritional Assessment was unchanged at follow‐up. ^3^Deteriorated nutrition: Participants whose nutritional status according to the Mini Nutritional Assessment was deteriorated at follow‐up.

This study was conducted using the Korean Frailty and Aging Cohort Study (KFACS), a multicentre longitudinal database from 2016 to 2023 [11]. KFACS covered 10 centres across rural, suburban and urban regions in Korea [11]. Participants recruited by each centre were 70–84 years old and were followed up every 2 years [11]. Among 3014 participants enrolled at baseline in 2016 and 2017 in this cohort, 170 participants were excluded for follow‐up loss, 914 were excluded for measurement loss for sarcopenia or nutritional status, and 269 who met the diagnostic criteria of sarcopenia at baseline were excluded. A total of 1661 individuals aged 70 or older and non‐sarcopenic at baseline were included in the final analysis (Figure 1). The study was approved by the Institutional Review Board (IRB) of the Clinical Research Ethics Committee, and all participants provided written informed consent (IRB number: 2015‐12‐103).

#### 2.1.1 Measurements of Mini Nutritional Assessment

The Mini Nutritional Assessment (MNA) was used to evaluate the nutritional status of participants at baseline and follow‐up using the full MNA questionnaire and standard guidelines. We chose the MNA full form because a previous cross‐sectional study by da Silva et al. demonstrated that malnutrition, as defined by the MNA, was associated with significantly increased odds of sarcopenia (OR 3.08, 95% CI 1.84–5.14) [11].

The MNA consists of 18 items that evaluate nutritional status across four domains: anthropometric, general, dietary and subjective assessment [12]. Anthropometric measurement included weight, height, body mass index (BMI), recent weight loss, mid‐arm circumference and calf circumference. General assessment included independent living, medication use, mobility and the presence of acute illness or psychological stress. Dietary assessment included the number of meals, dietary pattern, fluid intake and consumption of protein‐rich foods, fruits and vegetables, as well as chewing or swallowing difficulties that could affect food intake. Additionally, subjective assessment included self‐perception of nutritional and overall health status. The MNA has a total score of 30, and participants were split into three nutritional status groups based on their scores: normal nutrition (24–30 points), at risk of malnutrition (17–23.5 points) and malnutrition (< 17 points) [12]. Changes in nutritional status at baseline and at follow‐up were categorized as ‘improved’, ‘unchanged’ or ‘deteriorated’ based on transitions between these MNA groups.

#### 2.1.2 Assessment of Sarcopenia

Sarcopenia and severe sarcopenia were defined with adherence to Asian Working Group for Sarcopenia (AWGS) 2019 criteria [5]. Sarcopenia was defined as the presence of low muscle mass (dual‐energy x‐ray absorptiometry [DXA], < 7.0 kg/m^2^ in men and < 5.4 kg/m^2^ in women; bioelectrical impedance analysis (BIA), < 7.0 kg/m^2^ in men and < 5.7 kg/m^2^ in women) and low muscle strength (handgrip strength < 28 kg for men and < 18 kg for women) [5]. Severe sarcopenia was defined as the presence of low muscle mass, low muscle strength and low physical performance (Short Physical Performance Battery [SPPB] score ≤ 9, 6‐m walk < 1.0 m/s or 5‐time chair stand test ≥ 12 s) [5].

Appendicular skeletal muscle mass (ASM), muscle strength and physical performance were assessed using standardized protocols at designated centres to ensure consistency. ASM was measured using DXA (Hologic Horizon W [Hologic Inc., Bedford, MA, USA] for 852 participants, Lunar Prodigy [GE Healthcare Lunar, Madison, WI, USA] for 497 participants) and BIA (InBody 770 [InBody Co. Ltd., Seoul, Korea] for 312 participants), depending on the site equipment. Low muscle mass was determined based on ASM divided by height squared (kg/m^2^), in accordance with AWGS guidelines [11]. Muscle strength was assessed using the handgrip strength test. Grip strength of the dominant arm was measured twice, and the higher value was used for analysis. Physical performance was evaluated using the SPPB, which consists of a timed 4‐m gait speed test, a 5‐time sit‐to‐stand test and a set of standing balance tests. The standing balance test consisted of three positions: feet‐together, semi‐tandem and full‐tandem stances. Participants were timed for each position up to 10 s or until loss of balance. Each SPPB component was scored from 0 to 4, with total scores ranging from 0 to 12, representing an overall measurement of physical function [13].

The primary outcomes were the risks of new‐onset sarcopenia and severe sarcopenia, defined as meeting the respective AWGS 2019 diagnostic criteria at follow‐up among participants free of the condition at baseline.

#### 2.1.3 Statistical Analysis

Continuous variables were presented as the mean and standard deviation (SD) or median (interquartile range), whereas categorical variables were presented as number and percentage. One‐way analysis of variance (ANOVA) and the Kruskal–Wallis test were used for continuous variables, and the chi‐square test was used for categorical variables. To account for temporal changes in nutritional status during follow‐up, nutritional category (normal, at risk of malnutrition and malnourished) was modelled as a time‐varying covariate in the Cox proportional hazards regression. In sensitivity analyses, competing risk regression using the Fine and Gray subdistribution hazard model was also performed to account for death as a competing event [14]. Hazard ratios (HR), subdistribution hazard ratios (sHRs) and 95% confidence intervals (CIs) were derived for incident sarcopenia and severe sarcopenia. Model 1 was adjusted for age and sex. Model 2 was further adjusted for BMI, current smoking, current alcohol use, recent fall history and educational status. Model 3 was further adjusted for hypertension (HTN), diabetes mellitus (DM), dyslipidaemia (DL), congestive heart failure (CHF), renal disease, Korean Instrumental Activities of Daily Living (K‐IADL) and Korean Activities of Daily Living (K‐ADL). Kaplan–Meier (KM) survival analysis was used, and KM curves were presented with *p* values assessed by the log‐rank, Breslow and Tarone–Ware tests. All statistical analyses were conducted using SPSS Version 23 (IBM Corp., Armonk, NY) and R Version 4.4.1 (R Foundation for Statistical Computing, Vienna, Austria). The *p* values less than 0.05 were considered statistically significant.

## 3 Role of the Funding Source

Korea Disease Control and Prevention Agency and Seoul National University Bundang Hospital, the funding source of the study, had no role in the design of the study; collection, analysis or interpretation of the data; writing of the report; or decision to submit the report for publication.

## 4 Results

### 4.1 Baseline Characteristics

The baseline characteristics of participants, both overall and by change in nutritional status, are presented in Tables 1 and 2. Of the 1661 participants, 46.9% were male, and the mean age at baseline was 76.3 ± 3.6 years. The mean MNA score of all participants at baseline was 25.5 ± 2.6 points. Among them, 1284 (77.3%) had normal nutritional status, 362 (21.8%) were at risk of malnutrition, and 15 (0.9%) were malnourished at baseline. During follow‐up, nutritional status was ‘improved’ in 184 (11.1%) participants, ‘unchanged’ in 1232 (74.2%) and ‘deteriorated’ in 245 (14.8%). Participants with ‘improved’ nutritional status were more likely to have a lower baseline MNA score (21.6 ± 2.0 points) and lower physical parameters (ASM, hand grip strength, SPPB total score and TUG time; all *p* < 0.05) compared to those with ‘unchanged’ nutritional status. Prevalence of DM differed across groups (improved 22.3%, unchanged 20.0% and deteriorated 24.1%; *p* = 0.009), as well as renal disease (2.2%, 1.1%, 0.8%; *p* = 0.024) and osteoporosis (26.4%, 14.8% and 24.6%; *p* < 0.001). Frailty prevalence was higher in the improved group (22.6%) compared to the unchanged (7.4%) and deteriorated group (7.6%; *p* < 0.001). Educational level of high school or above was higher in the unchanged group (42.6%) compared to the improved (26.7%) and deteriorated group (36.5%; *p* < 0.001). Rural residence was more common in the improved group (33.2%) compared to the other groups (26.2% and 25.7%; *p* = 0.037).

**TABLE 1:** Baseline characteristics of the study population both overall and according to transition in MNA group over time.

	Total^a^	Improved nutrition	Unchanged nutrition	Deteriorated nutrition	*p* ^b^
*N*	1661	184	1232	245	
Age, years	76.3 ± 3.6	76.3 ± 3.6	76.1 ± 3.6	76.9 ± 3.7	0.005
Male	779 (46.9%)	63 (34.2%)	623 (50.6%)	93 (38.0%)	< 0.001
BMI, kg/m^2^	24.7 ± 2.9	23.9 ± 3.2	24.9 ± 3.0	24.3 ± 2.5	< 0.001
Current smoker	80 (4.8%)	59 (4.6%)	19 (5.3%)	2 (13.3%)	< 0.001
Social drinker	841 (50.8%)	692 (54.0%)	144 (39.9%)	5 (33.3%)	< 0.001
Height, cm	158.2 ± 8.4	155.7 ± 8.3	158.9 ± 8.2	156.7 ± 9.0	< 0.001
Weight, kg	62.0 ± 9.4	57.8 ± 8.0	63.1 ± 9.5	59.8 ± 8.6	< 0.001
Income > 2 million KRW	531 (33.8%)	36 (20.6%)	426 (36.5%)	69 (30.0%)	< 0.001
Follow‐up duration, days	1762 (1546–2141)	1748 (1462–2144)	1780 (1553–2141)	1738 (1538–2136)	0.279
K‐IADL	10 (10–13)	10 (10–11)	10 (10–13)	10 (10–13)	0.662
K‐ADL	7 (7–7)	7 (7–7)	7 (7–7)	7 (7–7)	< 0.001
Frailty (FRAIL)	147 (9.1%)	40 (22.6%)	89 (7.4%)	18 (7.6%)	< 0.001
Education level					
Primary school and below	695 (45.2%)	102 (61.8%)	477 (41.5%)	116 (52.3%)	< 0.001
Middle school	227 (14.8%)	19 (11.5%)	183 (15.9%)	25 (11.3%)	< 0.001
High school or above	615 (40.0%)	44 (26.7%)	490 (42.6%)	81 (36.5%)	< 0.001
Rural residence	444 (26.9%)	61 (33.2%)	320 (26.2%)	63 (25.7%)	0.037
Hypertension	973 (58.7%)	111 (60.3%)	714 (58.0%)	148 (60.7%)	0.524
Diabetes Mellitus	346 (20.8%)	41 (22.3%)	246 (20.0%)	59 (24.1%)	0.009
Dyslipidaemia	642 (39.2%)	73 (40.3%)	464 (38.2%)	105 (43.6%)	0.602
Heart failure	12 (0.7%)	3 (1.6%)	7 (0.6%)	2 (0.8%)	0.241
Renal disease	19 (1.1%)	4 (2.2%)	13 (1.1%)	2 (0.8%)	0.024
COPD	14 (0.8%)	1 (0.5%)	10 (0.8%)	3 (1.2%)	0.936
Cancer	31 (1.9%)	2 (1.1%)	22 (1.8%)	7 (2.9%)	0.628
Osteoporosis	289 (17.5%)	48 (26.4%)	181 (14.8%)	60 (24.6%)	< 0.001
Recent fall history^c^	386 (23.3%)	44 (24.0%)	277 (22.5%)	65 (26.5%)	0.010

Abbreviations: BMI, body mass index; COPD, chronic obstructive pulmonary disease; KRW, Korean won.

^a^ Continuous variables were presented as mean ± standard deviation or median (interquartile range); categorical variables were presented as frequency (percentage).

^b^ For continuous variables, *p* values were calculated by the one‐way analysis of variance, except for non‐normally distributed variables that were analysed using Kruskal–Wallis method. For categorical variables, *p* for trend was calculated by the chi‐square test.

^c^ Fall history within 12 months was considered recent.

**TABLE 2:** Baseline characteristics related to nutritional status and sarcopenia.

	Total^a^	Improved nutrition	Unchanged nutrition	Deteriorated nutrition	*p* ^b^
MNA, at baseline	25.5 ± 2.6	21.6 ± 2.0	26.0 ± 2.3	25.9 ± 1.7	< 0.001
Normal nutrition	1284 (77.3%)	0 (0%)	1048 (85.1%)	236 (96.3%)	< 0.001
At risk of malnutrition	362 (21.8%)	172 (93.5%)	181 (14.7%)	9 (3.7%)	< 0.001
Malnutrition	15 (0.9%)	12 (6.5%)	3 (0.2%)	0 (0%)	< 0.001
MNA, follow‐up	25.1 ± 2.7	25.3 ± 1.7	25.8 ± 2.4	21.6 ± 2.3	< 0.001
Normal nutrition	1223 (73.6%)	175 (95.1%)	1048 (85.1%)	0 (0%)	< 0.001
At risk of malnutrition	419 (25.2%)	9 (4.9%)	181 (14.7%)	229 (93.5%)	< 0.001
Malnutrition	19 (1.1%)	0 (0%)	3 (0.2%)	16 (6.5%)	< 0.001
New‐onset sarcopenia^c^	257 (15.5%)	25 (13.6%)	173 (14.0%)	59 (24.1%)	< 0.001
New‐onset severe sarcopenia^d^ ^s^	122 (8.4%)	15 (10.1%)	77 (7.0%)	30 (14.7%)	< 0.001
ASM, kg	16.7 ± 3.9	15.4 ± 3.4	17.0 ± 3.9	15.9 ± 3.8	< 0.001
ASM/height^2^, kg/m^2^	6.6 ± 1.1	6.3 ± 1.0	6.7 ± 1.1	6.4 ± 1.0	< 0.001
Hand grip strength, kg	26.9 ± 7.7	24.5 ± 7.0	27.5 ± 7.7	25.3 ± 7.6	< 0.001
SPPB total, points	12 (11–12)	11 (10–12)	12 (11–12)	11 (10–12)	0.001
SPPB gait speed, m/s	1.1 ± 0.2	1.1 ± 0.3	1.1 ± 0.2	1.1 ± 0.2	0.001
TUG, sec	9.9 (8.7–11.3)	10.1 (9.0–12.0)	9.8 (8.6–11.2)	9.9 (9.1–11.5)	0.001

Abbreviations: ASM, appendicular skeletal muscle mass; MNA, Mini Nutritional Assessment; SPPB, Short Physical Performance Battery; TUG, Timed Up and Go test.

^a^ Continuous variables were presented as mean ± standard deviation or median (interquartile range); categorical variables were presented as frequency (percentage).

^b^ For continuous variables, *p* values were calculated by the one‐way analysis of variance, except for non‐normally distributed variables that were analysed using Kruskal–Wallis method. For categorical variables, *p* for trend was calculated by the chi‐square test.

^c^ New‐onset sarcopenia was defined according to AGWS 2019 guidelines as low muscle mass (dual‐energy x‐ray absorptiometry, < 7.0 kg/m^2^ in men and < 5.4 kg/m^2^ in women; bioimpedance, < 7.0 kg/m^2^ in men and < 5.7 kg/m^2^ in women) and low muscle strength (handgrip strength < 28 kg for men and < 18 kg for women).

^d^ New‐onset severe sarcopenia was defined according to AWGS 2019 guidelines as low muscle mass, low muscle strength and low physical performance (Short Physical Performance Battery score ≤ 9).

The baseline prevalence of sarcopenia and low muscle mass varied according to the measurement device used. The prevalence of low muscle mass was significantly higher when measured with Hologic (64.8%) compared to GE Lunar (37.2%) and BIA (24.2%) (*p* < 0.001 for all pairwise comparisons). However, the differences were relatively less pronounced for the final diagnosis of sarcopenia (Hologic 17.6%, GE Lunar 12.7% and BIA 14.1%), although the difference between the two DXA systems remained statistically significant (*p* = 0.022 for Hologic vs. GE Lunar). Detailed prevalence data by device are presented in Table S1.

During a mean follow‐up of 4.8 years (1748 days), 257 (15.5%) participants developed sarcopenia, and 122 (8.4%) participants developed severe sarcopenia (Tables 1 and 2). Participants with ‘deteriorated’ nutritional status showed significantly higher prevalences of new‐onset sarcopenia and severe sarcopenia (24.1% and 14.7%, respectively) compared to those with ‘improved’ (13.6% and 10.1%) and ‘unchanged’ (14.0% and 7.0%) nutritional status.

### 4.2 Association of the Baseline Nutritional Status With Risk of New‐Onset Sarcopenia and Severe Sarcopenia

First, we examined the association between baseline nutritional status and risk of sarcopenia and severe sarcopenia, without excluding individuals with baseline sarcopenia (Table S2). Both ‘at risk of malnutrition’ and ‘malnutrition’ groups showed significantly increased risk of sarcopenia and severe sarcopenia at follow‐up, when compared to those with ‘normal nutrition’. To clarify the temporal association between malnutrition and new‐onset sarcopenia, participants with sarcopenia at baseline were excluded in subsequent tables (Table 3, Table 4 and Table S3).

**TABLE 3:** Multivariate analysis of the association between the baseline MNA group and the risk of new‐onset sarcopenia and severe sarcopenia.

	New‐onset sarcopenia^a^			New‐onset severe sarcopenia^b^		
	HR	95% CI	*p*	HR	95% CI	*p*
Unadjusted						
Normal nutrition	1.00	Reference		1.00	Reference	
At risk of malnutrition	1.44	1.09–1.90	0.011	1.92	1.31–2.80	0.001
Malnutrition	4.52	1.48–13.80	0.008	2.60	1.03–21.88	0.038
Model 1^c^						
Normal nutrition	1.00	Reference		1.00	Reference	
At risk of malnutrition	1.48	1.12–1.98	0.007	1.91	1.28–2.84	0.002
Malnutrition	5.94	2.40–14.69	< 0.001	3.25	1.05–27.66	0.041
Model 2^d^						
Normal nutrition	1.00	Reference		1.00	Reference	
At risk of malnutrition	1.23	0.90–1.68	0.200	1.47	1.02–2.46	0.039
Malnutrition	4.23	1.60–11.20	0.004	1.77	0.92–19.86	0.080
Model 3^e^						
Normal nutrition	1.00	Reference		1.00	Reference	
At risk of malnutrition	1.18	0.86–1.63	0.310	1.47	0.92–2.37	0.110
Malnutrition	5.18	1.86–14.43	0.002	2.64	1.00–32.40	0.048

Abbreviations: CI, confidence interval; sHR, subdistribution hazard ratio.

^a^ New‐onset sarcopenia was defined according to AGWS 2019 guidelines as low muscle mass (dual‐energy x‐ray absorptiometry, < 7.0 kg/m^2^ in men and < 5.4 kg/m^2^ in women; bioimpedance, < 7.0 kg/m^2^ in men and < 5.7 kg/m^2^ in women) and low muscle strength (handgrip strength < 28 kg for men and < 18 kg for women).

^b^ New‐onset severe sarcopenia was defined according to AGWS 2019 guidelines as low muscle mass, low muscle strength, and low physical performance (Short Physical Performance Battery score ≤ 9).

^c^ Model 1 was adjusted for age and sex.

^d^ Model 2 was further adjusted for BMI, smoking status, drinking status, recent fall history and educational level.

^e^ Model 3 was further adjusted for hypertension, diabetes mellitus, dyslipidaemia, chronic heart failure, renal disease, cancer, K‐IADL and K‐ADL. sHR was estimated using the Fine and Gray competing risk model, which accounts for the risk of death as a competing event.

**TABLE 4:** Multivariate analysis of the association between change in MNA group with risk of new‐onset sarcopenia and severe sarcopenia.

	New‐onset sarcopenia				New‐onset severe sarcopenia			
	Unadjusted	Model 1^a^	Model 2^b^	Model 3^c^	Unadjusted	Model 1	Model 2	Model 3
Total (*N* = 1661)								
Improved nutrition (*N* = 184)	1.51 [1.00–2.29]	1.53 [1.01–2.33]*	1.34 [0.86–2.08]	1.38 [0.89–2.15]	1.65 [0.91–2.97]	1.67 [0.93–3.02]	1.53 [0.83–2.83]	1.53 [0.83–2.83]
Unchanged nutrition (*N* = 1232)	1.00 (reference)				1.00 (reference)			
Deteriorated nutrition (*N* = 245)	2.14 [1.55–2.95]***	2.10 [1.53–2.90]***	1.81 [1.29–2.54]**	1.77 [1.25–2.51]**	2.40 [1.53–3.76]***	2.30 [1.46–3.61]***	2.09 [1.32–3.31]**	1.97 [1.23–3.16]**
Normal nutrition^d^ (*N* = 1284)								
Unchanged nutrition (*N* = 1048)	1.00 (reference)				1.00 (reference)			
Deteriorated nutrition (*N* = 236)	2.51 [1.73–3.62]***	2.52 [1.74–3.65]***	2.10 [1.41–3.13]***	2.07 [1.37–3.12]**	2.46 [1.42–4.27]**	2.38 [1.36–4.15]**	2.06 [1.16–3.67]*	1.95 [1.08–3.53]*
At risk of malnutrition^e^ (*N* = 362)								
Improved nutrition (*N* = 172)	0.98 [0.49–1.95]	1.06 [0.53–2.12]	0.76 [0.33–1.74]	0.83 [0.36–1.91]	1.29 [0.55–3.01]	1.48 [0.62–3.49]	1.33 [0.53–3.38]	1.45 [0.56–3.79]
Unchanged nutrition (*N* = 181)	1.00 (reference)				1.00 (reference)			
Deteriorated nutrition (*N* = 9)	1.38 [0.72–2.66]	1.34 [0.69–2.58]	1.52 [0.76–3.05]	1.48 [0.73–3.01]	2.02 [0.92–4.46]	2.08 [0.94–4.63]	2.06 [0.89–4.75]	2.01 [0.84–4.80]
Malnutrition^f^ (*N* = 15)								
Improved nutrition (*N* = 12)	0.84 [0.08–8.40]	0.89 [0.08–10.50]	1.60 [0.05–50.38]	1.00 [0.04–24.01]	n/a^g^			
Unchanged nutrition (*N* = 3)	1.00 (reference)				n/a			

^a^ Model 1 was adjusted for age and sex.

^b^ Model 2 was further adjusted for BMI, smoking status, drinking status, recent fall history and educational level.

^c^ Model 3 was further adjusted for hypertension, diabetes mellitus, dyslipidaemia, chronic heart failure, renal disease, cancer, K‐IADL and K‐ADL.

^d^ ‘Normal nutrition’ group was defined as an MNA score of 24 or greater.

^e^ ‘At risk of malnutrition’ group was defined as an MNA score of 17–23.5.

^f^ ‘Malnutrition’ group was defined as MNA score of less than 17. Changes in MNA scores were grouped as ‘improved’, ‘unchanged’ or ‘deteriorated’ nutrition based on transitions in MNA group over time. In ‘normal nutrition’ and ‘malnutrition’ groups, the ‘improved’ (*N* = 0) and ‘deteriorated’ nutrition groups (*N* = 0), respectively, were omitted due to the absence of participants.

^g^ In ‘malnutrition’ group, new‐onset of severe sarcopenia did not occur in the reference group (‘unchanged’), and thus, relative hazard ratio could not be compared in the ‘improved’ group. HR was estimated using the Cox proportional hazards regression with nutritional category (normal, at risk of malnutrition, and malnourished) modelled as a time‐varying covariate; unadjusted and adjusted hazard ratios with 95% confidence interval are shown in Table 4.

* *p* < 0.05.

** *p* < 0.01.

*** *p* < 0.001.

The KM curves by baseline MNA group demonstrated that participants in the ‘malnutrition’ and ‘at risk of malnutrition’ groups had higher risks of both new‐onset sarcopenia and severe sarcopenia (log‐rank *p* < 0.001; Figure S1a,b). In both the unadjusted and the fully adjusted model (Model 3), the ‘malnutrition’ group was associated with increased risks of both new‐onset sarcopenia (sHR 5.18, 95% CI 1.86–14.43) and severe sarcopenia (sHR 2.64, 95% CI 1.00–32.40), when compared to the ‘normal nutrition’ group (Table 3). In both the unadjusted model and the age‐ and sex‐adjusted model (Model 1), the ‘at risk of malnutrition’ group was associated with an increased risk of both new‐onset sarcopenia (sHR 1.48, 95% CI 1.12–1.98) and severe sarcopenia (sHR 1.91, 95% CI 1.28–2.84), when compared to the ‘normal nutrition’ group. However, in a further adjusted model (Model 2), the association remained only for severe sarcopenia, and in the fully adjusted model (Model 3), no significant association was observed.

In a subsequent exploratory analysis (Table S2), we examined the associations between baseline scores of individual MNA components and the risks of new‐onset sarcopenia and severe sarcopenia. Lower baseline scores in MNA components, specifically BMI (f), mode of feeding (n) and self‐view of nutritional status (o), were significantly associated with increased risk of sarcopenia: MNA‐f (HR 1.56, 95% CI 1.05–2.32), MNA‐n (HR 20.98, 95% CI 2.79–157.79) and MNA‐o (HR 1.53, 95% CI 1.09–2.15). Among these components, MNA‐f and MNA‐o were associated with increased risk at intermediate scores, but not at the lowest scores. Furthermore, lower baseline scores in MNA components, specifically food intake (a), BMI (f), mode of feeding (n) and self‐view of nutritional status (o), were significantly associated with increased risk of severe sarcopenia: MNA‐a (HR 1.69, 95% CI 1.08–2.66), MNA‐f (HR 1.78, 95% CI 1.13–2.80), MNA‐n (HR 46.10, 95% CI 5.68–374.33) and MNA‐o (HR 2.41, 95% CI 1.47–3.96). A consistent, score‐dependent trend was observed for the risk of severe sarcopenia across these components. Overall, a score of 0 in MNA‐n (mode of feeding) was associated with the highest risk of sarcopenia and severe sarcopenia among individual MNA components (HR 20.98, 95% CI 2.79–157.79 and HR 46.10, 95% CI 5.68–374.33, respectively).

When stratified by measurement device, the longitudinal association between baseline nutritional status and incident sarcopenia remained generally consistent (Table S4). In the Hologic group, which constituted the largest portion of the cohort, individuals ‘at risk of malnutrition’ had a significantly higher risk of new‐onset sarcopenia (HR 1.58, 95% CI 1.10–2.27) and severe sarcopenia (HR 2.50, 95% CI 1.52–4.14). Similarly, the BIA group showed a significantly increased risk for severe sarcopenia (HR 2.58 for ‘at risk’ and HR 9.52 for ‘malnutrition’). In the GE Lunar group, the point estimates for HRs were consistently greater than 1.0, suggesting a similar trend, although they did not reach statistical significance due to the limited number of incident events in this subgroup.

### 4.3 Association of the Change in Nutritional Status With Risk of New‐Onset Sarcopenia and Severe Sarcopenia

**FIGURE 2:**
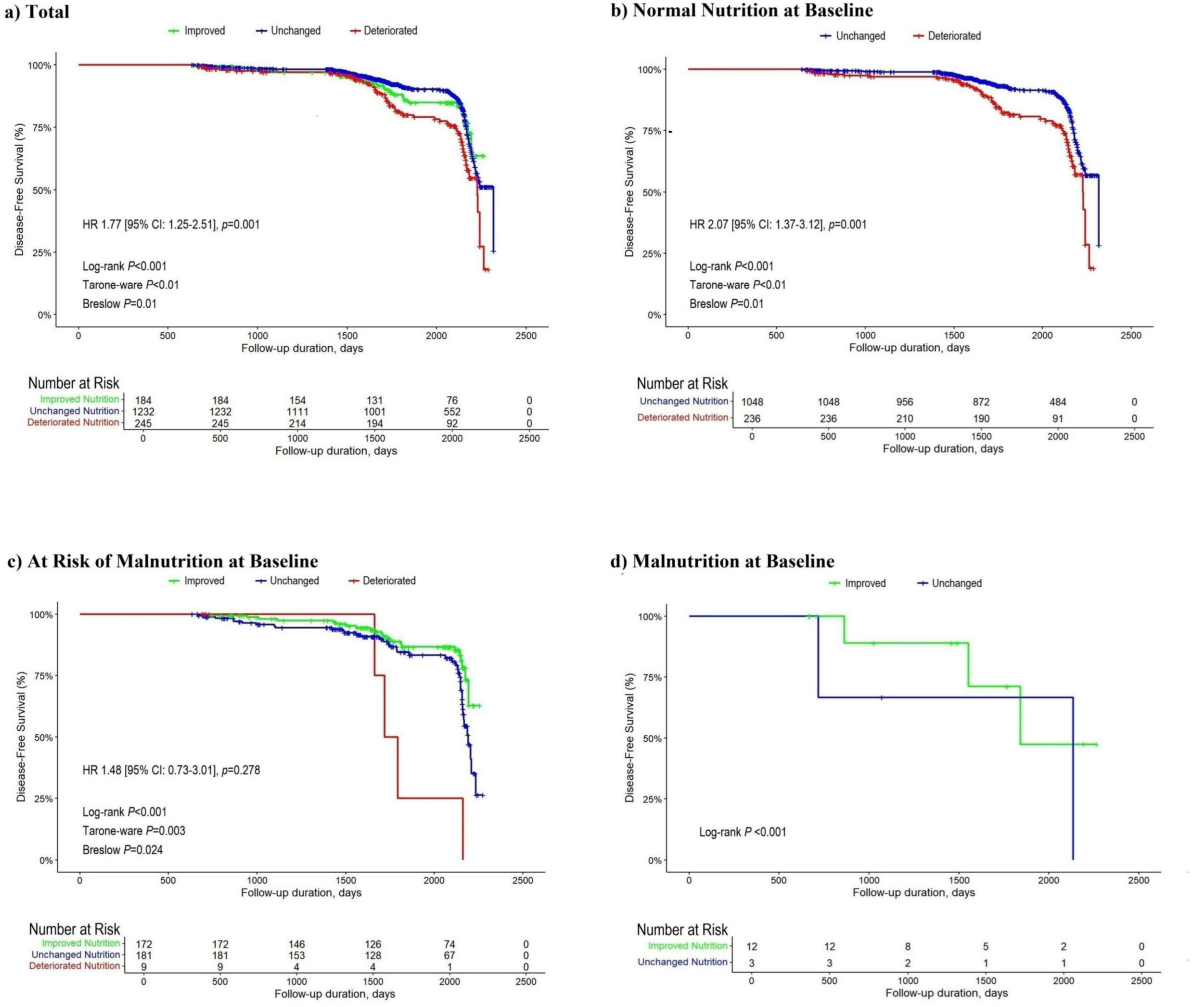
Kaplan–Meier curves for new‐onset sarcopenia according to change of nutritional status among total participants (a, *n* = 1661) and those with normal nutritional status (b, *n* = 1284), at risk of malnutrition (c, *n* = 362) or malnutrition (d, *n* = 15) at baseline. Compared to the unchanged group, the deteriorated group consistently showed higher risk across subgroups a through c. Hazard ratios, 95% confidence intervals and log‐rank *p* values are shown in each panel. Number at risk is indicated below each plot at 500‐day intervals.

KM survival analyses for new‐onset sarcopenia and severe sarcopenia, stratified by change in MNA group, were conducted in total participants (Figure 2a and Figure S2a, respectively) and within each baseline MNA group: ‘normal nutrition’ (Figure 2b and Figure S2b), ‘at risk of malnutrition’ (Figure 2c and Figure S2c) and ‘malnutrition’ (Figure 2d). Specifically, Figure 2 presents curves for new‐onset sarcopenia, and Figure S2 for severe sarcopenia. In both overall and subgroup analyses, ‘deteriorated’ nutritional status was associated with higher risk of both sarcopenia and severe sarcopenia compared to ‘unchanged’ nutritional status (log‐rank *p* < 0.001; Figure 2 and Figure S2).

Among participants with normal nutritional status at baseline, deterioration in nutritional status during follow‐up was associated with increased risks of sarcopenia (sHR 1.45, 95% CI 1.08–2.14) and severe sarcopenia (sHR 1.75, 95% CI 1.08–2.88), compared to those with ‘unchanged’ status (Table 4). Similarly, among those at risk of malnutrition at baseline, deterioration was associated with markedly higher risks of sarcopenia (sHR 4.79, 95% CI 1.39–16.59) and severe sarcopenia (sHR 9.24, 95% CI 2.01–42.35), compared to those with ‘unchanged’ status. In contrast, improvement in nutritional status was not significantly associated with lower risks of sarcopenia or severe sarcopenia compared to those with ‘unchanged’ status.

In time‐dependent Cox models with change in MNA group as a time‐dependent exposure, similar associations were observed among total participants and among those with baseline normal nutrition. Among total participants, deterioration was associated with higher risks of sarcopenia (HR 1.77, 95% CI 1.25–2.51) and severe sarcopenia (HR 1.97, 95% CI 1.23–3.16), compared to those with ‘unchanged’ status. Similarly, among those with baseline normal nutrition, deterioration was associated with higher risks of sarcopenia (HR 2.07, 95% CI 1.37–3.12) and severe sarcopenia (HR 1.95, 95% CI 1.08–3.53). In contrast, the time‐varying HRs for deterioration were not statistically significant among those at risk of malnutrition at baseline.

We further analysed the longitudinal association between changes in nutritional status and the development of sarcopenia, stratified by measurement device (Table S5). In the Hologic group, maintaining ‘unchanged’ nutrition was associated with a significantly higher risk of new‐onset severe sarcopenia compared to the ‘improved’ nutrition group (HR 2.29, 95% CI 1.17–4.47, *p* = 0.015). Similarly, in the BIA group, those with ‘deteriorated’ nutrition showed an increased risk for severe sarcopenia (HR 1.25, 95% CI 0.22–7.15), although the small number of events in this category limited the statistical significance. Across all devices, including GE Lunar, HR point estimates for unchanged or deteriorated nutrition generally remained above 1.0 for new‐onset sarcopenia, although they did not reach statistical significance.

## 5 Discussion

This study investigated the longitudinal association between changes in nutritional status and the risk of sarcopenia in community‐dwelling older adults. Baseline malnutrition was independently associated with a 3.6‐fold increased risk of sarcopenia and a 5.9‐fold increased risk of severe sarcopenia. In a component‐level exploratory analysis, lower baseline scores in specific MNA components, namely, food intake, BMI, mode of feeding and self‐view of nutritional status, were independently associated with increased risks of sarcopenia, with the greatest risk observed in participants with impaired feeding. Over a mean follow‐up period of 5.8 years, participants whose nutritional status deteriorated had a 1.5‐fold higher risk of sarcopenia and a 1.7‐fold higher risk of severe sarcopenia compared to those who maintained their nutritional status. However, improvement in nutritional status was not significantly associated with a reduced risk of sarcopenia. Overall, longitudinal changes in nutritional status were independent risk factors for sarcopenia, suggesting that maintaining adequate nutrition over time may be an important strategy for sarcopenia risk control in community‐dwelling older adults. Although nutritional deterioration was modelled as a time‐varying covariate preceding the subsequent assessment of sarcopenia, the 2‐year interval between measurements limits the ability to determine the exact biological sequence of events. Therefore, our findings should be interpreted as demonstrating a temporal and predictive association rather than establishing direct causality.

In the current study, participants with or at risk of malnutrition at baseline were more likely to develop sarcopenia and severe sarcopenia, which aligns with the results of prior research [8, 9, 15]. Gao et al. conducted a systematic review on the associated factors of sarcopenia in community‐dwelling older adults, and in this review, participants with malnutrition or at risk of malnutrition were three times more likely to be associated with sarcopenia than those without [9]. In addition, a longitudinal study conducted by Helen et al. showed that participants at risk of malnutrition at baseline were 9.3 times more likely to develop sarcopenia at a median follow‐up period of 4.2 years than those with normal nutritional status [8]. In this study, community‐dwelling adults aged over 60 years in Mexico City were analysed. The relative difference in hazard ratios between the Mexican cohort and our Korean population could be partly explained by differences in baseline age and nutritional status. The lower mean age of the Mexican cohort may have resulted in a lower initial prevalence of sarcopenia but a steeper relative increase in incident cases during follow‐up. More importantly, baseline nutritional status is likely to be a key determinant [16]. Malnutrition and risk of malnutrition were considerably more prevalent in the Mexican cohort compared to our Korean cohort, suggesting that poorer nutritional reserve may have contributed to both a higher absolute incidence and a greater relative hazard of sarcopenia. Furthermore, disparities in dietary protein intake, micronutrient adequacy and socio‐economic access to high‐quality nutrition across populations may influence susceptibility to muscle loss and progression to sarcopenia [17, 18]. Taken together, these findings indicate that nutritional status and dietary quality, rather than demographic factors alone, are critical determinants when interpreting population‐level differences in sarcopenia risk.

Longitudinal changes in nutritional status were associated with sarcopenia progression in the current study. Among individuals with normal nutrition at baseline, deterioration in nutritional status significantly increased the risk of both sarcopenia and severe sarcopenia in both conventional and time‐dependent Cox regression models using change in MNA as a time‐dependent covariate. This finding was more pronounced among those who were at risk of malnutrition at baseline; however, time‐dependent Cox regression did not reach statistical significance, most likely due to a small number of events and wider CIs. Regardless, this finding suggests that individuals with suboptimal baseline nutrition may be particularly vulnerable to sarcopenia progression. Additionally, the magnitude of skeletal muscle loss from a low‐calorie diet is significantly affected by baseline body composition, as well as nutrient supplementation and resistance training [19, 20]. Muscle wasting in participants with malnutrition or at risk of malnutrition may have been influenced by a habitual low‐calorie, low‐protein diet as well as accompanying risk factors such as inadequate supplementation and lack of exercise [21, 22]. Proposed biological mechanisms underlying this association include reduced muscle protein synthesis, increased inflammation and hormonal dysregulation, which collectively accelerate muscle loss [23, 24]. Previous studies have also suggested that nutrient deprivation, such as a lack of glucose or essential amino acids, accelerates muscle degradation via complex pathways including miRNA‐regulated autophagy [25]. This could explain why baseline nutritional status has a deteriorating impact on the incidence of sarcopenia. Future studies should investigate the mechanisms underlying the magnitude of sarcopenia progression in different subgroups.

Notably, the MNA is a validated assessment tool for malnutrition [5, 26]. A previous validation study of the MNA in community‐dwelling populations demonstrated a sensitivity of 97.9% and a specificity of 100% for the diagnosis of malnutrition [27]. Furthermore, a low MNA score, as an indicator of malnutrition risk, is an established risk factor for sarcopenia according to the AWGS 2019 guidelines [5]. In a study by da Silva et al., malnutrition defined by the MNA full form was associated with threefold increased odds of sarcopenia [11]. These findings underscore the clinical utility of the MNA in evaluating malnutrition and its associated risk of sarcopenia in community‐dwelling populations.

Individual components of MNA questionnaire, such as loss of appetite and low BMI, are shared with the Global Leadership Initiative of Malnutrition (GLIM) criteria for the diagnosis of malnutrition. In a previous study, significantly higher risks of developing sarcopenia (HR 3.23, 95% CI 1.73–6.05) and severe sarcopenia (HR 2.87, 95% CI 1.25–6.56) during a 4‐year follow‐up were observed in a malnutrition group defined by the GLIM criteria [22]. This finding supports the results of our exploratory analysis of individual MNA components, which demonstrated that a low baseline MNA score in food intake, BMI, mode of feeding or self‐perceived nutritional status was an independent predictor of sarcopenia progression. Self‐perceived malnutrition as an independent risk factor of sarcopenia is particularly novel. Future investigations should clarify the biological and behavioural mechanisms in how these specific MNA components may lead to muscle decline and test the potential role of targeted interventions on these domains.

This study has several strengths. First, it is among the few longitudinal studies focusing on the impact of nutritional status on the risk of sarcopenia, thereby offering insights that extend beyond those provided by cross‐sectional studies. Second, time‐dependent Cox regression demonstrated a consistently higher hazard of sarcopenia and severe sarcopenia with deteriorated nutritional status, aligning with our focus on the temporal effect of MNA change. Third, the use of standardized and validated assessment tools for both sarcopenia and nutrition strengthens the validity and comparability of measurements. Fourth, the multicentre design of the KFACS cohort, spanning 10 study centres across urban, suburban and rural regions nationwide, enhances the representativeness of community‐dwelling older adults. Lastly, adjustment for a wide range of confounding variables, previously reported in the literature in relation to sarcopenia, reinforces the robustness of our findings.

Despite these strengths, certain limitations must be acknowledged. First, although the MNA is a widely used nutritional assessment tool, it does not capture all aspects of nutritional adequacy, particularly specific micronutrient deficiencies that may contribute to sarcopenia. Second, the study used self‐reported dietary data, which may be subject to recall bias. Third, important confounders such as physical inactivity, chronic inflammation, hormonal dysregulation, mitochondrial dysfunction and cellular senescence were not included in this study [28, 29]. Nutritional deficits may interact with these pathways and exacerbate sarcopenia development but are best understood as a key modifiable contributor rather than the unique aetiological cause. Fourth, the average follow‐up duration of this study may not fully capture the effect of short‐term fluctuations in nutritional status. Fifth, as the study was conducted across multiple centres, different types of equipment (Hologic, GE Lunar and BIA) were used to assess body composition. We observed significant differences in the baseline prevalence of low muscle mass and sarcopenia depending on the device used, which is consistent with previous reports on systematic discrepancies between DXA systems [30, 31]. To mitigate this potential bias, we adjusted for the device type in all multivariate models and performed sensitivity analyses. These analyses confirmed that the longitudinal association between nutritional status and sarcopenia risk remained consistent across different devices, particularly in the Hologic and BIA subgroups. Sixth, the number of participants in the ‘malnutrition’ group was relatively small (*N* = 15), leading to wide CIs and limited statistical power in the subgroup analyses for this specific category. Therefore, the results for the malnutrition group should be interpreted as indicating a clinical trend rather than a precise risk estimate. Finally, the cohort consisted exclusively of Korean older adults, which may limit generalizability to populations with different ethnic, cultural or healthcare contexts, although previous research has shown that inadequate protein intake and malnutrition risk are common in older adults due to dietary patterns and socio‐economic disparities [32, 33]. Nonetheless, the core findings of this study, which underscore the importance of preventing nutritional decline in older adults, especially those at risk of malnutrition, may have broader relevance in context of global ageing.

In conclusion, this study demonstrates that not only baseline malnutrition but also longitudinal deterioration in nutritional status are independent predictors of incident sarcopenia and severe sarcopenia. However, as sarcopenia is a multifactorial condition, malnutrition should be interpreted as a modifiable contributor within a broader aetiological framework, rather than a singular causal factor. These findings underscore nutrition as a clinically actionable determinant rather than a background risk factor, highlighting the importance of incorporating regular nutritional screening into routine geriatric assessments to identify individuals at high risk before functional decline becomes irreversible. These results warrant further investigation into structured dietary interventions tailored to individuals with early nutritional decline to prevent or delay the onset of sarcopenia in older adults.

## Funding

This study was funded by grants from the Kyung Hee University (KHU‐20251278 in 2025) and Seoul National University Bundang Hospital.

## Conflicts of Interest

The authors declare no conflicts of interest.

## Supporting information


**Table S1:** Comparison of sarcopenia and low muscle mass prevalence according to measurement types.
**Table S2:** Multivariate analysis of risk of sarcopenia according to the baseline total MNA score^a^.
**Table S3:** Multivariate analysis of risk of new‐onset sarcopenia according to the baseline scores of individual MNA components^a^.
**Table S4:** Sensitivity analysis of baseline nutritional status according to measurement types.
**Table S5:** Sensitivity analysis of longitudinal nutritional changes according to measurement types.

**Figure S1:** Kaplan–Meier curves for new‐onset sarcopenia (a, *n* = 1661) and severe sarcopenia (b, *n* = 1449) according to baseline nutritional status among total participants. Compared to the ‘normal nutrition’ group, ‘malnutrition’ and ‘at risk of malnutrition’ groups showed higher risks of sarcopenia and severe sarcopenia. Hazard ratios, 95% confidence intervals and log‐rank *p* values are shown in each panel. Number at risk is indicated below each plot at 500‐day intervals.

**Figure S2:** Kaplan–Meier curves for new‐onset severe sarcopenia according to change of nutritional status among total participants (a, *n* = 1449) and those with normal nutritional status (b, *n* = 1147) or at risk of malnutrition (c, *n* = 293) at baseline. Compared to the unchanged group, the deteriorated group consistently showed higher risk across subgroups. Hazard ratios, 95% confidence intervals and log‐rank *p* values are shown in each panel. Number at risk is indicated below each plot at 500‐day intervals.
