# Supplementary material for: Longitudinal Deterioration in Nutritional Status Associated With Increased Risk of Sarcopenia in Community‐Dwelling Aged Adults: A Prospective Cohort Study
Source: J Cachexia Sarcopenia Muscle. 2026 Apr 1;17(2):e70270. doi: 10.1002/jcsm.70270 (PMC13042791; doi:10.1002/jcsm.70270)
Supplement: Supplementary file 1 — Table S1: Comparison of sarcopenia and low muscle mass prevalence according to measurement types. Table S2: Multivariate analysis of risk of sarcopenia according to the baseline total MNA scorea. Table S3: Multivariate analysis of risk of new‐onset sarcopenia according to the baseline scores of individual MNA componentsa. Table S4: Sensitivity analysis of baseline nutritional status according to measurement types. Table S5: Sensitivity analysis of longitudinal nutritional changes according to measurement types. [file JCSM-17-e70270-s002.docx]

**Supplementary Material**

**Table S1**

**Table S2** Multivariate analysis of risk of sarcopenia according to the baseline total MNA score

**Table S3** Multivariate analysis of risk of sarcopenia according to the baseline individual MNA scores

Table S4

Table S5

**Supplement Table S1. Comparison of sarcopenia and low muscle mass prevalence according to measurement types**

|  | Hologic (N=852) | GE Lunar (N=497) | BIA (N=312) | *p* (Hologic/Lunar) | *p* (Hologic/BIA) | *p* (Lunar/BIA) |
| --- | --- | --- | --- | --- | --- | --- |
| Sarcopenia, n (%) | 147 (17.5%) | 63 (12.7%) | 44 (14.1%) | **0.022** | 0.189 | 0.634 |
| Low mass, n (%) | 542 (64.7%) | 185 (37.2%) | 75 (24.2%) | **<0.001** | **<0.001** | **<0.001** |

Variables were presented as frequency (percentage). *P* values were calculated by the Chi-square test.

**Supplementary Table S2. Multivariate analysis of risk of sarcopenia according to the baseline total MNA score^a^**

|  | **Sarcopenia^b^** | | | **Severe sarcopenia^c^** | | |
| --- | --- | --- | --- | --- | --- | --- |
|  | **sHR** | **95% CI** | ***p-value*** | **sHR** | **95% CI** | ***p-value*** |
| **Unadjusted** |  | | | | | |
| **Normal nutritional status** | **1.00 (reference)** | | | | | |
| **At risk of malnutrition** | 1.59 | 1.23 - 1.80 | <0.001^***^ | 1.82 | 1.37 - 2.46 | <0.001^***^ |
| **Malnourished** | 3.65 | 1.79 – 6.99 | <0.001^***^ | 5.23 | 2.61 - 12.72 | <0.001^***^ |
| **Adjusted^d^** |  | | | | | |
| **Normal nutritional status** | **1.00 (reference)** | | | | | |
| **At risk of malnutrition** | 1.35 | 1.11 - 1.68 | <0.001^***^ | 1.81 | 1.35 - 2.41 | <0.001^***^ |
| **Malnourished** | 3.39 | 1.75 - 6.54 | <0.001^***^ | 4.62 | 1.58 – 11.57 | 0.001^**^ |

sHR, subdistribution hazard ratio; CI, confidence interval. ^a^ Multivariate cox regression analysis was performed in 1930 participants with or without sarcopenia at baseline to assess the risk of sarcopenia at follow-up. ^b^ Sarcopenia was defined according to AGWS 2019 as low muscle mass (dual-energy X-ray absorptiometry, <7.0 kg/m2 in men and <5.4 kg/m2 in women; bioimpedance, <7.0 kg/m2 in men and <5.7 kg/m2 in women) and low muscle strength (handgrip strength <28 kg for men and <18 kg for women). ^c^ Severe sarcopenia was defined according to AGWS 2019 guidelines as low muscle mass, low muscle strength, and low physical performance (Short Physical Performance Battery score ≤9). ^d^Adjusted for age and sex. sHR was estimated using the Fine and Gray competing risk model, which accounts for the risk of death as a competing event. *P<0.05, **P<0.01, ***P<0.001

**Supplementary Table S3. Multivariate analysis of risk of new-onset sarcopenia according to the baseline scores of individual MNA components^a^**

|  |  |  | **New onset sarcopenia ^a^** | | | | **New onset severe sarcopenia ^b^** | | | |
| --- | --- | --- | --- | --- | --- | --- | --- | --- | --- | --- |
| ***Individual***  ***MNA Score ^d^*** | |  | **Unadjusted** | | **Adjusted ^c^** | | **Unadjusted** | | **Adjusted** | |
|  |  | **n** | **HR [95% CI]** | ***p-value*** | **HR [95% CI]** | ***p-value*** | **HR [95% CI]** | ***p-value*** | **HR [95% CI]** | ***p-value*** |
| **a** | **2** | **1329** | 1.00 (reference) | | | | 1.00 (reference) | | | |
|  | **1** | **267** | 1.37 [0.79 - 2.37] | 0.265 | 1.57 [0.84 - 2.95] | 0.161 | **2.65 [1.41 - 4.99]** | **0.002^**^** | **3.08 [1.53 - 6.18]** | **0.002^**^** |
|  | **0** | **65** | **1.38 [1.01 - 1.90]** | **0.046^*^** | 1.24 [0.88 - 1.75] | 0.214 | **2.03 [1.32 - 3.13]** | **0.001^**^** | **1.69 [1.08 - 2.66]** | **0.022^*^** |
|  |  |  |  | | | |  | | | |
| **b** | **3** | **1381** | 1.00 (reference) | | | | 1.00 (reference) | | | |
|  | **2** | **206** | 1.16 [0.43 - 3.11] | 0.773 | - | - | 2.11 [0.67 - 6.66] | 0.205 | - | - |
|  | **1** | **46** | 1.69 [0.90 - 3.19] | 0.106 | - | - | 2.16 [0.95 - 4.95] | 0.068 | - | - |
|  | **0** | **28** | 1.26 [0.87 - 1.81] | 0.22 | - | - | 1.55 [0.95 - 2.54] | 0.082 | - | - |
|  |  |  |  | | | |  | | | |
| **c** | **2** | **1658** | 1.00 (reference) | | | | 1.00 (reference) | | | |
|  | **1** | **3** | 0.05 [0 - 1534148] | 0.733 | - | - | - | - | - | - |
|  | **0** | **0** | - | - | - | - | - | - | - | - |
|  |  |  |  | | | |  | | | |
| **d** | **2** | **1518** | 1.00 (reference) | | | | 1.00 (reference) | | | |
|  | **0** | **143** | 1.07 [0.68 - 1.68] | 0.782 |  |  | 0.99 [0.50 - 1.96] | 0.984 | - | - |
|  |  |  |  | | | |  | | | |
| **e** | **2** | **1644** | 1.00 (reference) | | | | 1.00 (reference) | | | |
|  | **1** | **1** | 1.39 [0.44 - 4.33] | 0.574 | - | - | 1.72 [0.24 - 12.33] | 0.589 | - | - |
|  | **0** | **16** | - | - | - | - | - | - | - | - |
|  |  |  |  | | | |  | | | |
| **f ^e^** | **3** | **1185** | 1.00 (reference) | | | | 1.00 (reference) | | | |
|  | **2** | **311** | 1.63 [0.67 - 3.97] | 0.283 | 1.11 [0.39 - 3.14] | 0.851 | 1.48 [0.36 - 6.05] | 0.585 | 0.96 [0.21 - 4.36] | 0.962 |
|  | **1** | **142** | **1.76 [1.22 - 2.54]** | **0.002^**^** | **1.56 [1.05 - 2.32]** | **0.027^*^** | **1.79 [1.04 - 3.08]** | **0.035^*^** | 1.51 [0.86 - 2.64] | 0.152 |
|  | **0** | **23** | 1.24 [0.90 - 1.70] | 0.184 | 1.32 [0.94 - 1.85] | 0.106 | **1.78 [1.17 - 2.70]** | **0.007^**^** | **1.78 [ 1.13 - 2.80]** | **0.012^*^** |
|  |  |  |  | | | |  | | | |
| **g** | **1** | **1356** | 1.00 (reference) | | | | 1.00 (reference) | | | |
|  | **0** | **305** | 0.99 [0.73 - 1.34] | 0.95 | - | - | 1.04 [0.67 -1.61] | 0.859 | - | - |
|  |  |  |  | | | |  | | | |
| **h** | **1** | **926** | 1.00 (reference) | | | | 1.00 (reference) | | | |
|  | **0** | **735** | **1.52 [1.19 - 1.94]** | **0.001^**^** | 1.25 [0.92 - 1.69] | 0.151 | **1.68 [1.18 - 2.40]** | **0.004^**^** | 1.37 [0.89 - 2.10] | 0.153 |
|  |  |  |  | | | |  | | | |
| **i** | **1** | **1652** | 1.00 (reference) | | | | 1.00 (reference) | | | |
|  | **0** | **9** | 0.94 [0.13 - 6.68] | 0.948 | - | - | 0.05 [0 - 7541] | 0.621 | - | - |
|  |  |  |  | | | |  | | | |
| **j** | **2** | **1512** | 1.00 (reference) | | | | 1.00 (reference) | | | |
|  | **1** | **146** | - | - | - | - | - | - | - | - |
|  | **0** | **3** | 1.04 [0.68 -1.59] | 0.85 | - | - | 1.39 [0.81 - 2.39] | 0.234 | - | - |
|  |  |  |  | | | |  | | | |
| **k** | **1** | **161** | 1.00 (reference) | | | | 1.00 (reference) | | | |
|  | **0.5** | **601** | **1.74 [1.05 - 2.87]** | **0.031^*^** | 1.57 [0.92 - 2.68] | 0.097 | 1.72 [0.86 - 3.44] | 0.123 | 1.34 [0.65 - 2.73] | 0.425 |
|  | **0** | **899** | 1.59 [0.95 - 2.66] | 0.081 | 1.39 [0.81 - 2.39] | 0.233 | 1.30 [0.63 - 2.68] | 0.479 | 1.11 [0.53 - 2.30] | 0.79 |
|  |  |  |  | | | |  | | | |
| **l** | **1** | **1383** | 1.00 (reference) | | | | 1.00 (reference) | | | |
|  | **0** | **278** | 1.02 [0.75 - 1.40] | 0.884 | - | - | 1.18 [0.77 - 1.83] | 0.45 | - | - |
|  |  |  |  | | | |  | | | |
| **m** | **1** | **1170** | 1.00 (reference) | | | | 1.00 (reference) | | | |
|  | **0.5** | **428** | 0.87 [0.44 - 1.70] | 0.677 | - | - | 1.32 [0.57 - 3.02] | 0.518 | - | - |
|  | **0** | **63** | 1.16 [0.89 - 1.51] | 0.284 | - | - | 1.37 [0.94 - 2.00] | 0.105 | - | - |
|  |  |  |  | | | |  | | | |
| **n** | **2** | **1648** | 1.00 (reference) | | | | 1.00 (reference) | | | |
|  | **1** | **2** | 0.40 [0.06 - 2.88] | 0.366 | 0.33 [0.05 - 2.39] | 0.27 | 0.95 [0.13 - 6.78] | 0.945 | 0.63 [0.09 - 4.99] | 0.681 |
|  | **0** | **11** | **12.79 [1.77 - 92.24]** | **0.011^*^** | **20.98 [2.79 -157.79]** | **0.003^**^** | **26.58 [3.61 - 195.65]** | **0.001 ^**^** | **46.10 [5.68 - 374.33]** | **<0.001^***^** |
|  |  |  |  | | | |  | | | |
| **o** | **2** | **1071** | 1.00 (reference) | | | | 1.00 (reference) | | | |
|  | **1** | **251** | **1.44 [1.07 - 1.93]** | **0.015^*^** | **1.53 [1.09 - 2.15]** | **0.013^*^** | **1.86 [1.21 - 2.84]** | **0.004 ^**^** | **1.94 [1.20 - 3.13]** | **0.007^**^** |
|  | **0** | **339** | 1.14 [0.80 - 1.62] | 0.474 | 1.33 [0.90 - 1.95] | 0.149 | **1.77 [1.11 - 2.80]** | **0.016 ^*^** | **2.41 [1.47 - 3.96]** | **<0.001^***^** |
|  |  |  |  | | | |  | | | |
| **p** | **2** | **306** | 1.00 (reference) | | | | 1.00 (reference) | | | |
|  | **1** | **945** | 1.45 [0.95 - 2.21] | 0.085 | - | - | 1.48 [0.85 - 2.59] | 0.166 | - | - |
|  | **0.5** | **139** | 1.62 [1.00 - 2.64] | 0.051 | - | - | 1.39 [0.71 - 2.70] | 0.336 | - | - |
|  | **0** | **271** | 1.12 [0.79 - 1.60] | 0.528 | - | - | 0.79 [0.49 - 1.28] | 0.336 | - | - |
|  |  |  |  | | | |  | | | |
| **q** | **1** | **1651** | 1.00 (reference) | | | | 1.00 (reference) | | | |
|  | **0.5** | **8** | - | - | - | - | - | - | - | - |
|  | **0** | **2** | **4.87 [2.01 - 11.83]** | **<0.001^***^** | 2.34 [0.84 - 6.57] | 0.105 | 2.22 [0.31 - 15.94] | 0.427 | 0.69 [0.09 - 5.15] | 0.72 |
|  |  |  |  | | | |  | | | |
| **r** | **1** | **1512** | 1.00 (reference) | | | | 1.00 (reference) | | | |
|  | **0** | **149** | **1.92 [1.34 - 2.74]** | **<0.001^***^** | 1.09 [0.68 - 1.73] | 0.731 | **2.21 [1.34 - 3.65]** | **0.002^**^** | 1.03 [0.54 - 1.95] | 0.939 |

^a^ New-onset sarcopenia was defined according to AGWS 2019 guidelines as low muscle mass (dual-energy X-ray absorptiometry, <7.0 kg/m2 in men and <5.4 kg/m2 in women; bioimpedance, <7.0 kg/m2 in men and <5.7 kg/m2 in women) and low muscle strength (handgrip strength <28 kg for men and <18 kg for women). ^b^ New-onset severe sarcopenia was defined according to AGWS 2019 guidelines as low muscle mass, low muscle strength, and low physical performance (Short Physical Performance Battery score ≤9). ^c^ Model was adjusted for age, sex, body mass index, smoking status, drinking status, recent fall history, educational level, hypertension, diabetes mellitus, dyslipidemia, chronic heart failure, renal disease, K-IADL, and K-ADL.

^d^ Individual components of MNA assess for decreased food intake (a), recent weight loss (b), mobility (c), psychological stress or acute disease (d), neuropsychological problems (e), body mass index (BMI) (f), independent living (g), number of drugs (h), pressure sores or skin ulcers (i), number of meals (j), protein intake (k), fruit and vegetable intake (l), fluid consumption (m), mode of feeding (n), self-view of nutritional status (o), self-view of health status (p), mid-arm circumference (q), and calf circumference (r). ^e^ Baseline BMI was excluded from Model 3 in the Cox regression analysis of MNA-f to avoid overadjustment.

^*^*P*<0.05, ^**^*P*<0.01, ^***^*P*<0.001

**Supplement Table S4. Sensitivity analysis of baseline nutritional status according to measurement types**

|  | **New-onset sarcopenia^a^** | | | **New-onset severe sarcopenia^b^** | | |
| --- | --- | --- | --- | --- | --- | --- |
|  | **sHR** | **95% CI** | ***p-value*** | **sHR** | **95% CI** | ***p-value*** |
| **Hologic** | | | | | | |
| **Normal nutrition** | 1.00 | Reference | | 1.00 | Reference | |
| **At risk of malnutrition** | **1.58** | **[1.10 – 2.27]** | **0.014** | **2.5** | **[1.52 – 4.14]** | **< 0.001** |
| **Malnutrition** | 2.08 | [0.29 – 14.98] | 0.467 | 13.45 | [0.83 – 216.5] | 0.068 |
| **GE Lunar** | | | | | | |
| **Normal nutrition** | 1.00 | Reference | | 1.00 | Reference | |
| **At risk of malnutrition** | 1.35 | [0.76 – 2.40] | 0.301 | 0.95 | [0.39 – 2.32] | 0.906 |
| **Malnutrition** | **2.65** | [0.62 – 11.28] | 0.187 | 0 | N/A | 0.999 |
| **BIA** | | | | | | |
| **Normal nutrition** | 1.00 | Reference | | 1.00 | Reference | |
| **At risk of malnutrition** | 1.21 | [0.60 – 2.41] | 0.596 | **2.58** | **[1.11 – 5.99]** | **0.027** |
| **Malnutrition** | 6.22 | [0.74 – 52.40] | 0.091 | **9.52** | **[1.18 – 76.5]** | **0.034** |

sHR, subdistribution hazard ratio; CI, confidence interval. ^a^Sarcopenia was defined according to AGWS 2019 as low muscle mass (dual-energy X-ray absorptiometry, <7.0 kg/m2 in men and <5.4 kg/m2 in women; bioimpedance, <7.0 kg/m2 in men and <5.7 kg/m2 in women) and low muscle strength (handgrip strength <28 kg for men and <18 kg for women). ^b^Severe sarcopenia was defined according to AGWS 2019 guidelines as low muscle mass, low muscle strength, and low physical performance (Short Physical Performance Battery score ≤9). Adjusted for age, sex, BMI, smoking status, drinking status, recent fall history, educational level, hypertension, diabetes mellitus, dyslipidemia, chronic heart failure, renal disease, cancer, K-IADL, and K-ADL. sHR was estimated using the Fine and Gray competing risk model, which accounts for the risk of death as a competing event.

**Supplement Table S5. Sensitivity analysis of longitudinal nutritional changes according to measurement types**

|  | **New-onset sarcopenia^a^** | | | **New-onset severe sarcopenia^b^** | | |
| --- | --- | --- | --- | --- | --- | --- |
|  | **HR** | **95% CI** | ***p-value*** | **HR** | **95% CI** | ***p-value*** |
| **Hologic** | | | | | | |
| **Improved Nutrition** | 1.00 | Reference | | 1.00 | Reference | |
| **Unchanged Nutrition** | 1.09 | [0.62 – 1.91] | 0.758 | **2.29** | **[1.17 – 4.47]** | **0.015** |
| **Deteriorated Nutrition** | 1.63 | [0.73 – 3.65] | 0.231 | 1.84 | [0.70 – 4.86] | 0.219 |
| **GE Lunar** | | | | | | |
| **Improved Nutrition** | 1.00 | Reference | | 1.00 | Reference | |
| **Unchanged Nutrition** | 1.75 | [0.84 – 3.64] | 0.137 | 0.75 | [0.17 – 3.26] | 0.706 |
| **Deteriorated Nutrition** | 1.22 | [0.38 – 3.92] | 0.738 | 0.44 | [0.05 – 4.31] | 0.485 |
| **BIA** | | | | | | |
| **Improved Nutrition** | 1.00 | Reference | | 1.00 | Reference | |
| **Unchanged Nutrition** | 0.35 | [0.08 – 1.49] | 0.156 | 0.7 | [0.16 – 3.07] | 0.636 |
| **Deteriorated Nutrition** | 0.92 | [0.17 – 4.98] | 0.925 | 1.25 | [0.22 – 7.15] | 0.803 |

sHR, subdistribution hazard ratio; CI, confidence interval. ^a^Sarcopenia was defined according to AGWS 2019 as low muscle mass (dual-energy X-ray absorptiometry, <7.0 kg/m2 in men and <5.4 kg/m2 in women; bioimpedance, <7.0 kg/m2 in men and <5.7 kg/m2 in women) and low muscle strength (handgrip strength <28 kg for men and <18 kg for women). ^b^Severe sarcopenia was defined according to AGWS 2019 guidelines as low muscle mass, low muscle strength, and low physical performance (Short Physical Performance Battery score ≤9). Adjusted for age, sex, BMI, smoking status, drinking status, recent fall history, educational level, hypertension, diabetes mellitus, dyslipidemia, chronic heart failure, renal disease, cancer, K-IADL, and K-ADL. sHR was estimated using the Fine and Gray competing risk model, which accounts for the risk of death as a competing event.
