# Supplementary figures and images for: Longitudinal Deterioration in Nutritional Status Associated With Increased Risk of Sarcopenia in Community‐Dwelling Aged Adults: A Prospective Cohort Study
Source: J Cachexia Sarcopenia Muscle. 2026 Apr 1;17(2):e70270. doi: 10.1002/jcsm.70270 (PMC13042791; doi:10.1002/jcsm.70270)

### a) Sarcopenia

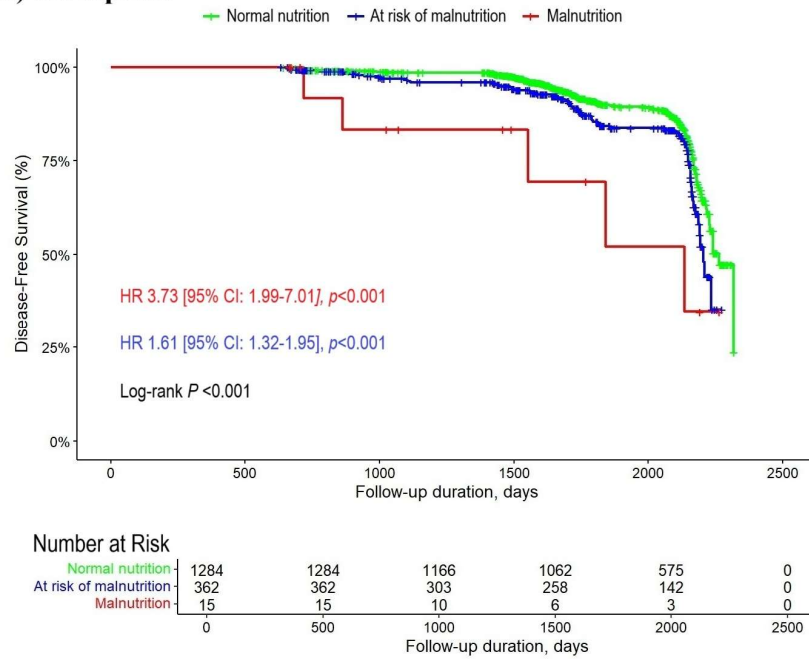

### b) Severe Sarcopenia

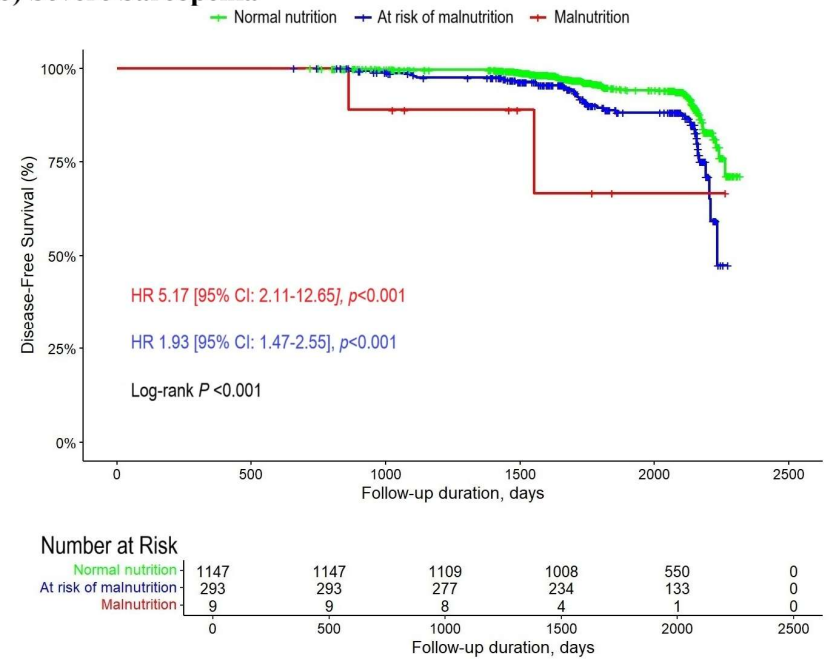

Supplement: Supplementary file 2 — Figure S1: Kaplan–Meier curves for new‐onset sarcopenia (a, n = 1661) and severe sarcopenia (b, n = 1449) according to baseline nutritional status among total participants. Compared to the ‘normal nutrition’ group, ‘malnutrition’ and ‘at risk of malnutrition’ groups showed higher risks of sarcopenia and severe sarcopenia. Hazard ratios, 95% confidence intervals and log‐rank p values are shown in each panel. Number at risk is indicated below each plot at 500‐day intervals. [file JCSM-17-e70270-s001.pdf]
