# Supplementary material for: Longitudinal Deterioration in Nutritional Status Associated With Increased Risk of Sarcopenia in Community‐Dwelling Aged Adults: A Prospective Cohort Study
Source: J Cachexia Sarcopenia Muscle. 2026 Apr 1;17(2):e70270. doi: 10.1002/jcsm.70270 (PMC13042791; doi:10.1002/jcsm.70270)

a) Total

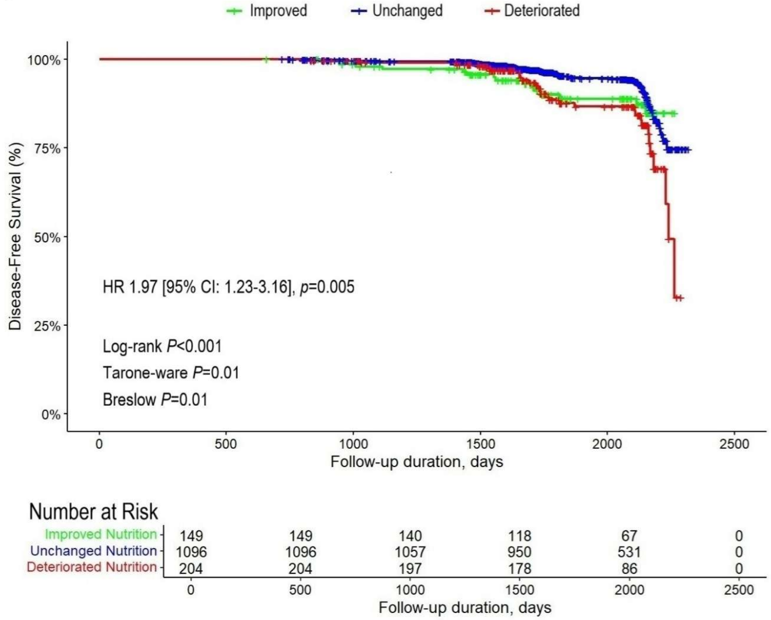

b) Normal Nutrition at Baseline

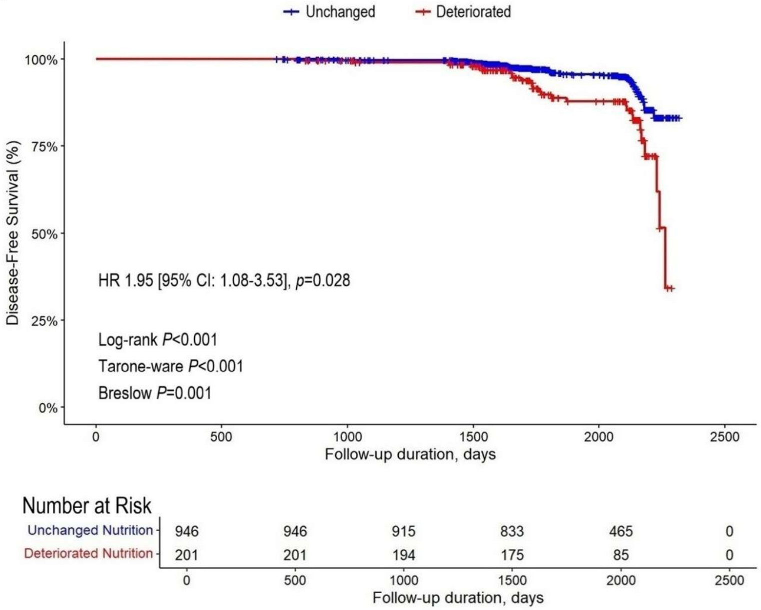

c) At Risk of Malnutrition at Baseline

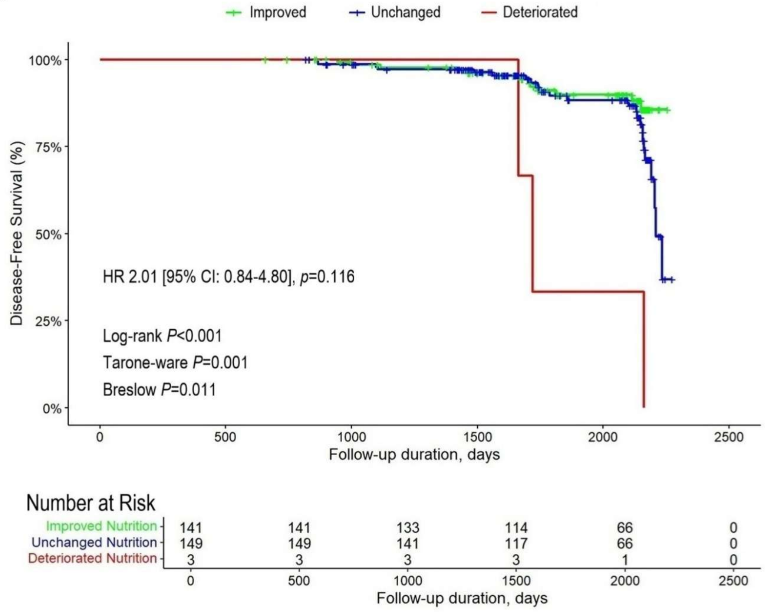

Supplement: Supplementary file 3 — Figure S2: Kaplan–Meier curves for new‐onset severe sarcopenia according to change of nutritional status among total participants (a, n = 1449) and those with normal nutritional status (b, n = 1147) or at risk of malnutrition (c, n = 293) at baseline. Compared to the unchanged group, the deteriorated group consistently showed higher risk across subgroups. Hazard ratios, 95% confidence intervals and log‐rank p values are shown in each panel. Number at risk is indicated below each plot at 500‐day intervals. [file JCSM-17-e70270-s003.pdf]
